# An In-Depth Analysis of a Piece of Shit: Distribution of *Schistosoma mansoni* and Hookworm Eggs in Human Stool

**DOI:** 10.1371/journal.pntd.0001969

**Published:** 2012-12-20

**Authors:** Stefanie J. Krauth, Jean T. Coulibaly, Stefanie Knopp, Mahamadou Traoré, Eliézer K. N'Goran, Jürg Utzinger

**Affiliations:** 1 Department of Epidemiology and Public Health, Swiss Tropical and Public Health Institute, Basel, Switzerland; 2 University of Basel, Basel, Switzerland; 3 Centre Suisse de Recherches Scientifiques en Côte d'Ivoire, Abidjan, Côte d'Ivoire; 4 Unité de Formation et de Recherche Biosciences, Université de Cocody, Abidjan, Côte d'Ivoire; Case Western Reserve University School of Medicine, United States of America

## Abstract

**Background:**

An accurate diagnosis of helminth infection is important to improve patient management. However, there is considerable intra- and inter-specimen variation of helminth egg counts in human feces. Homogenization of stool samples has been suggested to improve diagnostic accuracy, but there are no detailed investigations. Rapid disintegration of hookworm eggs constitutes another problem in epidemiological surveys. We studied the spatial distribution of *Schistosoma mansoni* and hookworm eggs in stool samples, the effect of homogenization, and determined egg counts over time in stool samples stored under different conditions.

**Methodology:**

Whole-stool samples were collected from 222 individuals in a rural part of south Côte d'Ivoire. Samples were cut into four pieces and helminth egg locations from the front to the back and from the center to the surface were analyzed. Some samples were homogenized and fecal egg counts (FECs) compared before and after homogenization. The effect of stool storing methods on FECs was investigated over time, comparing stool storage on ice, covering stool samples with a water-soaked tissue, or keeping stool samples in the shade.

**Principal Findings:**

We found no clear spatial pattern of *S. mansoni* and hookworm eggs in fecal samples. Homogenization decreased *S. mansoni* FECs (p = 0.026), while no effect was observed for hookworm and other soil-transmitted helminths. Hookworm FECs decreased over time. Storing stool samples on ice or covered with a moist tissue slowed down hookworm egg decay (p<0.005).

**Conclusions/Significance:**

Our findings have important implications for helminth diagnosis at the individual patient level and for epidemiological surveys, anthelmintic drug efficacy studies and monitoring of control programs. Specifically, homogenization of fecal samples is recommended for an accurate detection of *S. mansoni* eggs, while keeping collected stool samples cool and moist delayed the disintegration of hookworm eggs.

## Author Summary

An accurate diagnosis of parasitic worm (helminth) infections is important for adequate patient treatment and disease control programs. Helminth eggs in human stool samples are used as an indicator of infection intensity and morbidity. However, little is known about the exact distribution of helminth eggs in stool samples. Homogenization has been suggested to improve the diagnostic accuracy. Hookworm eggs disintegrate over time, which makes their detection challenging in epidemiological surveys. We determined the location of helminth eggs in entire stool samples from 222 individuals in Côte d'Ivoire. We also investigated whether homogenization has an effect on the detection of eggs, and determined egg counts over time in stool samples stored on ice, covered with a moist tissue, or kept in the shade. No clear pattern of helminth egg distribution was found in human stool samples. Homogenization resulted in more accurate egg counts of the blood fluke *Schistosoma mansoni*, while it did not affect other helminths. Keeping stool samples on ice or covered with a wet tissue slows down the disintegration of hookworm eggs. Our findings have important implications for individual patient management and the design and implementation of epidemiological surveys and helminth disease control programs.

## Introduction

Although schistosomiasis and soil-transmitted helminthiasis affect hundreds of millions of people and account for more than 40% of the global burden due to neglected tropical diseases, parasitic worm infections are often still neglected [1]–[3]. An accurate diagnosis is necessary for adequate patient treatment, a deeper understanding of the epidemiology of helminthiases, assessment of anthelmintic drug efficacy, and for monitoring the community-effectiveness of control programs [4], [5].

The Kato-Katz technique is the current method of choice for the diagnosis of *Schistosoma mansoni*, *Schistosoma japonicum*, and soil-transmitted helminths in epidemiological studies [6]–[8]. However, the Kato-Katz technique has several shortcomings. First, there is a lack of sensitivity, with light infection intensities particularly prone to be missed [9]–[12]. Second, there is considerable intra- and inter-specimen variation of helminth egg distribution and aggregation in feces [10], [11], [13]–[16]. Interestingly though, contradicting findings have been reported on the spatial distribution of helminth eggs in human stool samples [16], [17]. For example, two studies reported significantly higher *S. japonicum* fecal egg counts (FECs) on the surface compared to the center [16], [17]. For *S. mansoni*, however, no significant differences in FECs have been found [18], [19]. Similarly, no differences have been observed in the distribution of eggs of the two soil-transmitted helminths, *Ascaris lumbricoides* and *Trichuris trichiura* in human stool samples [20]. To date only two studies examined helminth egg distribution along the length axis of the stool and both focused on schistosome species [16], [21]. Of note, in these studies, sample sizes were very small (≤11 individuals), and hence the reported results have to be interpreted with caution. Third, a time delay from stool production to processing the specimens in the laboratory is influencing the sensitivity of helminth diagnosis, particulary for hookworm [22], [23].

Homogenization of fecal material has been suggested as one way to overcome intra-specimen variation of helminth egg counts [22],[23]. However, the effect of homogenization on helminth FECs has yet to be determined. In fact, we could identify only two studies that examined the effect of homogenization on the variability of the distribution of *S. japonicum*, *T. trichiura*, and *A. lumbricoides* eggs within entire stool samples [17], [20]. Another study merely states a suggestion about the effect of homogenizing based on findings about the similarity of FECs from the center and surface of stool [21]. Studies on soil-transmitted helminths concluded that homogenizing by stirring would not overcome the influence of intra-specimen variation of helminth egg location, while the study on *S. japonicum* showed that variation decreased after stirring [17], [20].

Yet, the aforementioned features are likely to affect the performance of all diagnostic methods for which sampling of fecal material is involved. We therefore aimed to investigate the nature of intra-specimen variation of helminth egg distribution, to examine whether there is any effect of homogenization for the detection and quantification of helminth eggs in stool samples, and to monitor FECs over time in stool samples stored on ice, covered with a water-soaked tissue, or kept in the shade. Emphasis was placed on *S. mansoni* and hookworm infections. Results from our study might be of direct relevance for stool sampling and laboratory work-up in diagnostic centers, helminth epidemiological surveys, anthelmintic drug efficacy evaluation, and control programs.

## Methods

### Ethics Statement

The study protocol was approved by the institutional research commission of the Swiss Tropical and Public Health Institute (Swiss TPH, Basel, Switzerland). Ethical approval was granted by the ethics committees of Basel (EKBB, reference no. 377/09) and Côte d'Ivoire (reference no. 1993 MSHP/CNER). District health and village authorities, study participants and parents/guardians of individuals aged <18 years, were informed about the purpose, procedures, and potential risks and benefits of the study. Written informed consent was obtained from participants or the parents/guardians of minors before the collection of the first stool sample. Participation was voluntary and there were no further obligation for those who withdrew from the study. All results were coded and treated confidentially.

At the end of the study, all participants who provided a stool sample for the parasitological investigation were treated with albendazole (single 400 mg oral dose) and praziquantel (single 40 mg/kg oral dose) free of charge, irrespective of their helminth infection status.

### Study Area

The study was carried out in Azaguié, a small town in the district of Agboville in south Côte d'Ivoire. Azaguié is situated approximately 40 km north of Abidjan, the economic capital of Côte d'Ivoire. Soil-transmitted helminthiasis and schistosomiasis are highly endemic [24]–[26]. There are four main seasons in the area, a long dry season from December to March, the main rainy season between April and mid-July, a short dry season from mid-July to mid-September, and a short rainy season from mid-September to November. The average temperature is 27°C and the annual precipitation is 1,200–1,500 mm.

### Field Procedures

**Figure 1:**
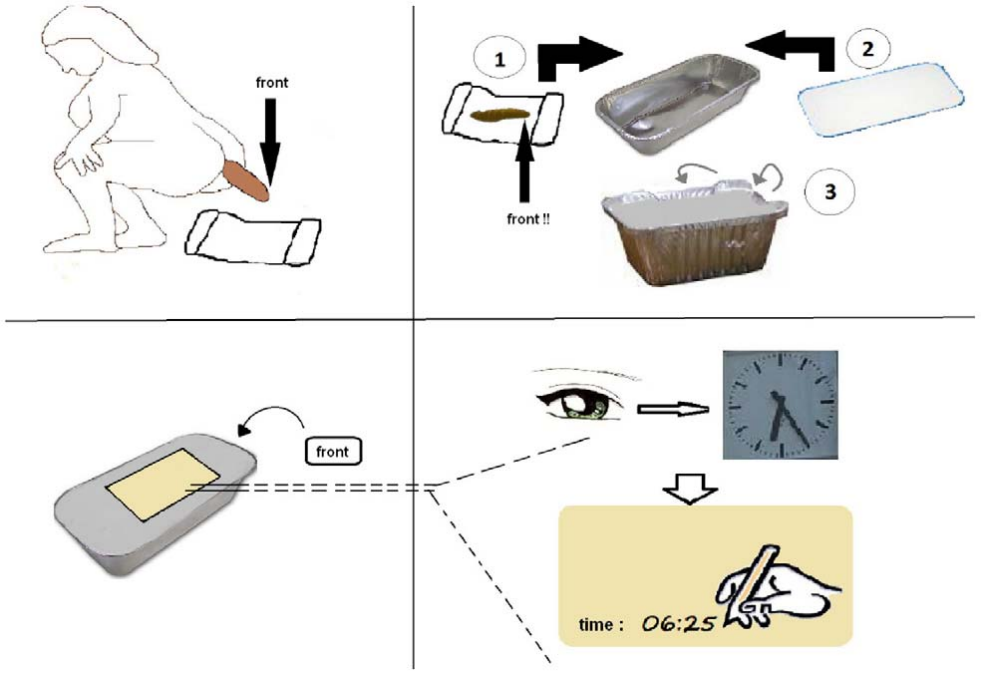
Instruction form on how to collect whole-stool samples for the study.

The study was conducted in September and October 2010. Once local authorities and nearby schools had been informed, the research team invited all interested people of the area to come to an information session. After participants gave their written informed consent, they were supplied with an aluminum ‘take away box’, together with a cover-lid, a sheet of aluminum foil, a sticker, and an instruction form explaining how to collect the stool sample (Figure 1). Participants were invited to place their fresh morning feces directly on the aluminum foil, while trying to keep it in a long, straight shape. Subsequently, participants were asked to wrap the stool into the foil without breaking it and place it into the ‘take-away box’. The box was closed and a sticker put on the box to mark the front of the stool (defined as that part of the stool that had left the bowel first). Mushy samples, where no front could be indicated, were not marked. Finally, participants were asked to note the exact time of defecation (hh∶mm) onto the lid and return the sample to the laboratory at the Azaguié health center, where the diagnostic work-up took place.

### Laboratory Procedures

In a first step, stool specimens were categorized into one of five consistency categories: (i) sausage-shaped (equivalent to type 3 on the Bristol Stool Chart (BSC) [27]); (ii) sausage-shaped-but-lumpy (type 2 BSC); (iii) sausage-shaped-but-soft (type 4 BSC); (iv) lumpy (type 1 BSC); and (v) mushy (types 5–7 BSC).

**Figure 2:**
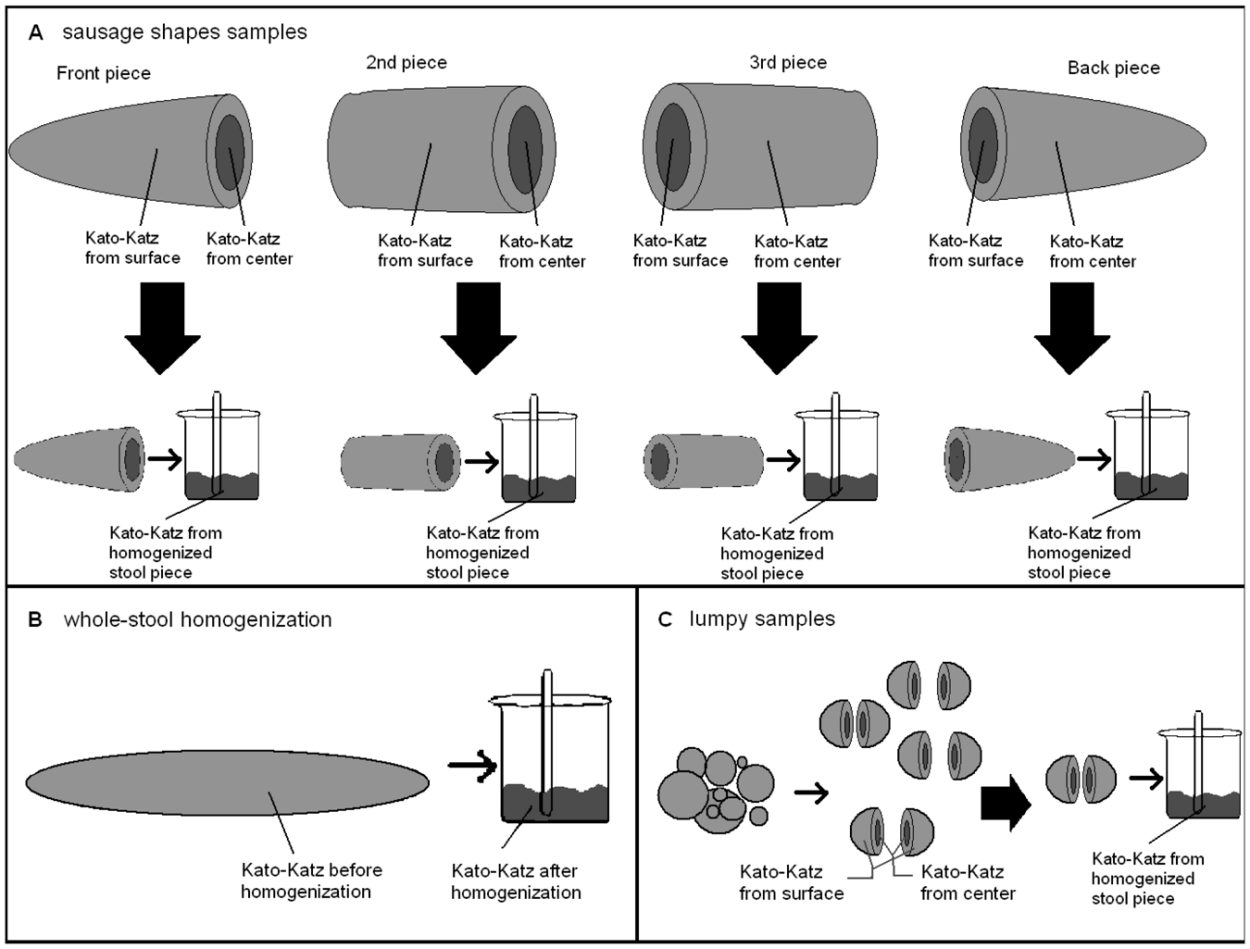
Processing of stool samples according to consistency including whole-stool homogenization. *Sausage-shaped-but-soft samples were processed like in (A) without taking samples from the center.

Next, samples of each category were randomly allocated either to the assessment of whole-stool homogenization or to the assessment of helminth egg distribution in stool by drawing lots. Figure 2 shows how samples designated for assessment of helminth egg distribution in stool were further stratified according to their consistency. In brief, sausage-shaped samples, including sausage-shaped-but-lumpy samples, were cut into four equally sized pieces along the length axis (named front, second, third, and back piece). Care was taken that no fecal material was transferred from one piece to another. For each piece of stool, one Kato-Katz thick smear (using standard 41.7 mg templates [28]) was prepared from the center and a second one from the surface. Center samples were obtained by breaking open the stool piece without transferring fecal material from the surface region into the center, and by subsequently taking a small piece of stool from the center using a fresh spatula. Surface material was taken by scraping or cutting-off feces from the outer layer of the stool with a spatula, not invading deeper than 2–3 mm into the inside of the stool. Subsequently, the remaining part of the stool piece was put into a plastic cup and thoroughly homogenized by stirring with a plastic tongue spatula by two different laboratory assistants for at least 1 min each. A third Kato-Katz thick smear was then prepared from the thoroughly homogenized fecal material.

Sausage-shaped-but-soft stool samples were cut into four equally long pieces as described above. However, these samples were too soft to properly take stool from the center and the surface. Hence, a first Kato-Katz thick smear was prepared from any unspecified region of the piece and a second one after the homogenization had been completed.

Lumpy samples were not cut. Sufficiently big lumps were chosen and Kato-Katz thick smears were prepared from the center, surface, and the homogenized lump, as described above.

Mushy samples of types 5 and 6 BSC were always allocated to the assessment of whole-stool homogenization, as they were not suitable for any of the other processing schemes. Very liquid samples (type 7 BSC) were excluded from the different diagnostic approaches as they were not suitable for any processing. However a single Kato-Katz thick smear was prepared from these samples to identify the participant's helminth infection status.

From all samples allocated to the assessment of whole-stool homogenization, prior to homogenization, a first Kato-Katz thick smear was prepared by taking material from any unspecified region of sample, as mostly done in routine analysis. The rest of the whole-stool sample was then transferred into a plastic cup and thoroughly homogenized by stirring with a plastic tongue spatula by two different laboratory assistants for at least 1 min each. A second Kato-Katz thick smear was prepared from the homogenized whole-stool sample.

To avoid confounding due to the time delay from production to examination of the different stool pieces, we established four distinctive examination time points, each lasting 2 h. At each time point, one stool piece per sample was randomly chosen to be examined using the aforementioned methodology by drawing lots. Using this approach, we assured that each piece of each stool sample was examined at a randomly chosen, but predefined time point of the day and we avoided introducing any systematic error into the analysis of egg distribution. Our rationale was as follows. If stool sections would have been analyzed in a systematic way (i.e., analyzing the front first and the back last) and assuming that FEC might decline over time, we might have observed a spatial egg distribution pattern with eggs declining from the front to the back. However, in reality, this observation would be due to the effect of helminth egg decay over time rather than real spatial egg distribution. By randomly choosing the time point of analysis for each piece, the potential confounder (i.e., time) is removed. Additionally, this procedure allowed us to monitor for potential change in FECs over time.

Independent of their processing schemes, approximately one third of all samples were stored in a box kept on ice, a third was stored being covered with a tissue soaked with tap water, and the remaining third was stored without any additional preservation efforts but placed in the shade outside the laboratory.

We adhered to World Health Organization (WHO) guidelines for Kato-Katz thick smear preparation and examination [29], [30]. In brief, Kato-Katz thick smears were read by an experienced laboratory technician in a systematic way within a maximum of 60 min after preparation. The number of helminth eggs was recorded for each species separately. Ten percent of all slides were subjected to quality control and checked for internal consistency. In case of conflicting results, all Kato-Katz thick smears of that individual where re-read and the results of the second reading were used for analysis. Additionally, all data recording sheets were checked for internal consistency.

### Statistical Analysis

Data were double entered by a single person into EpiInfo™ version 3.4.1 (EpiInfo 2007), checked for internal consistency and then analyzed with STATA version 10.1 (Stata 2009). A stool sample was considered positive for a specific helminth species if at least one egg was found in any of the slides examined. Egg counts derived from individual Kato-Katz thick smears were multiplied by a factor of 24 to obtain a standardized value of eggs per gram of stool (EPG). Helminth species specific EPG values of each individual were categorized into infection intensities according to cut-off values provided by WHO [31].

A random effects negative binomial regression (NBR) model was performed for all assessments to check for the interaction of FECs between the different pieces or locations. Additionally, the Wilcoxon (WXN) signed rank test was used for categorical data whenever two categories were compared, and the Kendall's coefficient of concordance (KCC) was used to show the degree of agreement between FECs obtained in each stool part. A KCC value of 1 defines total agreement and a KCC value of 0 indicates no agreement.

For the assessment of differences in FECs from formed stool and mushy stool, a non-overlapping 95% confidence interval (CI) indicates significance. For the assessment of the effect of homogenization, mushy stool samples were excluded because we assumed that they already were homogeneous.

## Results

### Study Population and Sample Allocation

**Figure 3:**
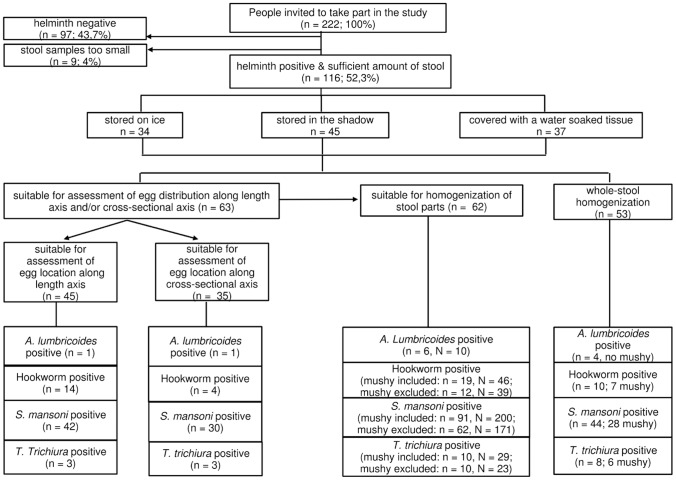
Flowchart detailing inclusion and exclusion of study participants and use for the various analysis including helminth prevalences. n, number of whole-stool samples; N, number of stool pieces.

Stool samples were collected from a total of 222 individuals aged 5–52 years with a median age of 12 years. Among them, 125 (56.3%) were infected with at least one helminth species. However, nine helminth-positive participants were excluded from the statistical analysis because their samples were of insufficient amount to be examined with more than a single Kato-Katz thick smear. Of the remaining 116 stool samples, 34 were stored on ice, 45 in a box kept in the shade, and 37 covered with a water-soaked tissue. The whole-stool homogenization assay was performed with 53 stool samples and 63 samples were allocated to the assessment of helminth egg distribution in different locations of the stool. Details of the exact number of samples allocated to each examination procedure are shown in Figure 3.

### Prevalence of *S. mansoni* and Soil-Transmitted Helminths

Among the 222 study participants, 102 (45.9%) were infected with *S. mansoni*, 23 (10.4%) were positive for *T. trichiura*, 21 (9.5%) for hookworm, and seven (3.2%) for *A. lumbricoides*. Additionally, two participants (0.9%) were infected with the tapeworm *Hymenolepis nana* (Table 1), but no further analysis was made for this parasite. Infections with two and three different helminth species were found in 25 (11.3%) and seven (3.2%) participants, respectively. With the exception of *S. mansoni* (35.5% of the positive individuals having ≥400 EPG) and hookworm (4.8% of the positive individuals having ≥4,000 EPG), heavy infections did not occur. Most soil-transmitted helminth infections were light (92.5%) and some were moderate (5.7%).

**Table 1:** Helminth prevalences and infection intensities among 125 helminth-positive study participants, from Azaguié, Côte d'Ivoire, in 2010.

	No.(%) of infected	Infection intensity		
		Light	Moderate	Heavy
*Schistosoma mansoni*	102 (45.9)	40 (39.2%)	26 (25.5%)	36 (35.5%)
*Trichuris trichiura*	23 (10.4)	23 (100%)	0	0
Hookworm	21 (9.5)	18 (85.7%)	2 (9.5%)	1 (4.8%)
*Ascaris lumbricoides*	7 (3.2)	6 (85.7%)	1 (14.3%)	0
*Hymenolepis nana*	2 (0.9)	2 (100%)	0	0
Any helminth	125 (100)			
Double infection	25 (20.0)			
Triple infection	7 (5.6)			

### Stool Parameters

Stool was produced between 05:30 and 11:10 hours (median: 08:35 hours) and collected between 07:52 and 11:19 hours (median: 09:05 hours). On average, nine samples were collected each day (range: 2–11 samples). One third of all samples (33.8%) were sausage-shaped, 28.8% were mushy, 25.2% were sausage-shaped-but-soft, 7.7% were lumpy, and 4.5% were sausage-shaped-but-lumpy (Table 2). An interesting observation worth mentioning is that whole-stool samples were more likely very small when participants were infected with *T. trichiura* compared to participants infected with other helminth species (OR = 1.88). The delay from stool production to the start of the Kato-Katz preparations in the laboratory was, on average, 120 min (range 20–300 min).

**Table 2:** Number of stool samples in each consistency category by infection status (any helminth infection), from 222 study participants from Azaguié, Côte d'Ivoire, in 2010.

	Total (% of total stool samples)	No. of infected (% of all infected stool samples)	No. of non infected (% of all non infected stool samples)
**n**	**222**	**125** *	**97**
Sausage like	75 (33.8%)	39 (33.6%)	33 (34.0%)
Sausage shaped but lumpy	10 (4.5%)	5 (4.3%)	4 (4.1%)
Sausage like but soft	56 (25.2%)	33 (28.4%)	22 (22.7%)
Lumpy	17 (7.7%)	4 (3.5%)	10 (10.3%)
Mushy	64 (28.8%)	35 (30.2%)	28 (28.9%)

* Nine helminth-positive stool samples were too small to allocate them to any consistency category.

### Helminth Egg Distribution along the Length Axis of the Stool

In general, FECs of all helminth species in formed stool from type 1–3 BSC were significantly higher than in soft stool from type 4–7 BSC, with a combined mean FEC ratio of 947∶447 EPG (95% CI: 698–1,196 EPG *versus* 323–579 EPG). *S. mansoni*-positive stool samples (*n* = 42), showed no significant differences in FECs between different locations along the length axis (z_NBR_ = −1.60; p = 0.111). There was an agreement of W_KCC_ = 0.92 between the ranks of FECs in all four stool pieces (p<0.001).

In hookworm-infected individuals (*n* = 14), FECs in the front piece of the stool sample were significantly higher than FECs in the back piece (z_NBR_ = −3.11, p = 0.002). In *T. trichiura*-positive stool samples (*n* = 3), FECs in the back piece were significantly higher than in the front (z_NBR_ = 2.63, p = 0.008). There was only one *A. lumbricoides*-positive sample among the samples dedicated to the assessment of helminth egg location, and hence no comparisons were made for this helminth species.

Mean FECs of *S. mansoni*, hookworms and *T. trichiura* in the different stool pieces along the length axis of the stool, can be seen in Table 3.

**Table 3:** Mean FECs (expressed as EPGs) in different stool pieces along the length axis of whole-stool samples, among 59 helminth-positive participants from Azaguié, Côte d'Ivoire, in 2010.

	n samples	Front piece (95% CI)	2^nd^ piece (95% CI)	3^rd^ piece (95% CI)	Back piece (95% CI)
*S. mansoni*	42	656 (372–940)	670 (346–995)	616 (349–883)	670 (275–841)
Hookworm	14	986 (149–1823)	396 (0–1040)	422 (0–1191)	267 (0–713)
*T. trichiura*	3	0	0	8 (0–24)	56 (15–98)

### Helminth Egg Distribution on the Surface and Center of the Stool

For *S. mansoni*-positive individuals, no difference in FECs between the surface and the center of the stool samples was found (z_NBR_ = −1.88; p = 0.060; *n* = 95). When eliminating pseudoreplication and analyzing only one randomly selected piece per stool sample, the effect was even stronger, but lacked statistical significance (z_NBR_ = 0.85; p = 0.276; *n* = 30). Similarly, we did not detect any difference in FECs between the surface or center for hookworm-positive (z_NBR_ = −0.12, p = 0.902; *n* = 4) and *T. trichiura*-positive stool samples (z_NBR_ = 1.85 p = 0.064; *n* = 3).

Mean FECs of *S. mansoni*, hookworms and *T. trichiura* in the surface and the center of the stool, can be seen in Table 4.

**Table 4:** Mean eggs per gram (EPG) values in surface and center of stool samples, among 102 helminth-positive participants from Azaguié Côte d'Ivoire, in 2010.

	n	Center (95% CI)	Surface (95% CI)
*S. mansoni*	95	41 (29–52)	32 (23–42)
Hookworm	4	90 (19–160)	90 (0–191)
*T. trichiura*	3	7 (0–18)	3 (1–4)

### Effect of Homogenization on Helminth Egg Distribution

*S. mansoni* FECs decreased after homogenization of stool samples, irrespectively of whether whole-stool samples (z_WXN_ = 2.14, p = 0.032; *n* = 16) or single pieces of a stool sample were homogenized (z_WXN_ = 2.23, p = 0.026; *n* = 62). The homogenization had an effect on the *S. mansoni* infection intensity category of 13 individuals: a change from heavy (≥400 EPG) to moderate (100–399 EPG), from moderate to light (1–99 EPG), and from light to moderate intensity was each observed in 4 people. A change from light to heavy infection intensity was found in one individual.

With regard to soil-transmitted helminths, no difference in FECs before and after homogenization was found, neither for whole-stool samples (hookworm, p = 0.166, *n* = 10; *T. trichiura*, p = 0.655, *n* = 8; *A. lumbricoides*, p = 0.456, *n* = 4), nor for the homogenization of one randomly chosen piece per sample (hookworm, p = 0.745, *n* = 12; *T. trichiura*, p = 0.217, *n* = 10; *A. lumbricoides*, p = 0.917, *n* = 6).

Intra-sample variance of FECs decreased significantly after homogenization for *S. mansoni* (z_WXN_ = 4.53, p<0.001, *n* = 40), but not for soil-transmitted helminths (hookworm, p = 0.655, *n* = 9; *T. trichiura*, p = 0.288, *n* = 8; *A. lumbricoides*, p = 0.953, *n* = 2).

Stratified by infection intensity, we found that the reduction of variance in *S. mansoni* FECs after homogenization only occurred with heavy (p = 0.001, *n* = 17) and moderate infection intensities (p = 0.008, *n* = 12), but not with light infection intensities (p = 0.588, *n* = 11). Additionally, the rate of Kato-Katz thick smears initially found positive that “changed” to negative after homogenization (14 out of 64, 21.8%) is nearly equal to the rate of Kato-Katz thick smears that were initially found negative and “changed” to positive after homogenization (17 out of 64, 26.7%) (both p>0.5).

### Effect of Stool Storing and Examination of Time Delays on FECs

The ranks of FECs from *S. mansoni* did not differ between the different time points of stool examination, irrespective of whether samples were stored on ice (W_KCC_ = 0.87, p<0.001, *n* = 30), covered with a water-soaked tissue (W_KCC_ = 0.87, p<0.001, *n* = 35), or kept in the shade (W_KCC_ = 0.91, p<0.001, *n* = 42).

In hookworm-positive stool samples, we observed a significant decline of FECs over time for samples stored in the shade (z_NBR_ = −2.82, p = 0.005, *n* = 12), although the agreement of the ranks of FECs determined at different time points was W_KCC_ = 0.73 (p = 0.001). Thus, we performed an additional WXN test for the stool pieces that were analyzed first and the pieces that were analyzed last. The test confirmed a significant decline of FECs over time (z_WXN_ = 2.45, p = 0.014).

If samples were stored on ice, no significant changes in FECs of hookworm were found over time (W_KCC_ = 0.84, p = 0.005, *n* = 12). Samples kept humid showed no significant change of ranks of FECs over time (W_KCC_ = 0.87, p<0.001, *n* = 12).

For *T. trichiura*-positive samples, no difference in ranks of FECs between the measured time points were observed, neither for samples stored on ice (W_KCC_ = 0.61, p = 0.080, *n* = 3), nor covered with a water-soaked tissue (W_KCC_ = 0.60, p<0.001, *n* = 4), nor kept in the shade (W_KCC_ = 0.67, p = 0.003, *n* = 11). Due to the small sample size for *A. lumbricoides*-positive samples in the assessment of FECs over time, no comparison was made.

## Discussion

An accurate diagnosis of helminth infection is of pivotal importance for adequate individual patient management, epidemiological surveys, intervention studies, and the monitoring of helminth control programs [4], [32]. Despite its relevance for diagnosis, the spatial distribution of helminth eggs in entire stool samples, and the effect of storage, homogenization and time delays of FECs have been assessed only rudimentarily. Here, we present an in-depth analysis of a “piece of shit”, detailing the spatial distribution of *S. mansoni* and soil-transmitted helminth eggs within whole stool samples, and determined the effect of different stool storage approaches, homogenization and time delays from stool production to laboratory processing on helminth egg detection.

The distribution of *S. mansoni* eggs without any clear pattern in whole stool speciments observed in our study corroborates with findings reported more than 40 years ago [21]. Our results, however, are in conflict with another study that reported a clear trend with *S. japonicum* eggs being predominantly located in the beginning and the surface of the stool [16]. These contradictions might either be due to a “real” difference between the egg distribution patterns of *S. mansoni* and *S. japonicum*, or due to the very low sample size (n = 5) in the study of Yu and colleagues [16]. The authors speculated that differences in the spatial egg distribution of the two schistosome species might be due to the different location of the adult worms harboring in the mesenteric veins [16].

The predominance of observing hookworm eggs in the front of stool specimens revealed in our study cannot be generalized since, by chance, 10 among 14 front pieces of hookworm-positive stool samples were examined at the first time-point, and 11 of the back pieces either at time-points 3 or 4. The decay of hookworm eggs as a function of time delays between stool production and processing revealed in our as well as previous studies [22] is the likely explanation of this observation. Hence, additional research is warranted to shed new light on the distribution of hookworm eggs along the main axis of entire stool specimens. Moreover, our finding that *T. trichiura* eggs are mainly located in the end pieces of stool is hampered due to the low size of positive samples. However, if future studies reveal or confirm that hookworm and *T. trichiura* eggs are mainly located in the front or end piece of the stool, this will have important implications on stool sampling procedures that should be taken into consideration for helminth diagnosis.

Our observation of no difference in egg location between the center and the surface of stool samples for any of the helminth species encountered in the current study area is in line with previous studies [18], [19]. However, although sample sizes for soil-transmitted helminth-positive stool samples in the present study are larger than in previous investigations, they remain small, particularly for *A. lumbricoides* and *T. trichiura*, and hence, results have to be interpreted with care. Unless future studies will show distinctive patterns of helminth eggs within stool samples, we feel that the current practice of scoping small pieces of stool anywhere from a stool specimen remains a valid method.

Homogenization of stool samples reduced intra-sample variance of *S. mansoni* FECs. Hence, we recommend that stool sample be homogenized prior to diagnosis to enhance the accuracy of S. mansoni egg detection. This recommendation should be considered for standard operating procedures for *S. mansoni* diagnosis.

Our study also confirms that, while time and storing method has no effect on FECs of *S. mansoni* and *T. trichiura*, hookworm FECs significantly decline with increasing time delays between stool production and laboratory processing of fecal material. Additionally we show – to our knowledge for the first time – that storing feces on ice or covered with a wet tissue can delay hookworm egg disintegration over time, which has important ramification for field procedures in any project involving hookworm diagnosis. Notably, hookworm eggs have been reported to be sensitive to dry conditions [33]. During our fieldwork we indeed observed that stool samples dried out quickly, especially on the surface. Hence, we recommend keeping stool samples humid pending laboratory work-up, best using a moist tissue, and regard this as a simple, cheap, and practical procedure to avoid hookworm egg disintegration. In other studies, refrigerating stool has been applied for the same purpose [22] and also in our study storing of fecal samples on ice was an effective strategy to delay the hookworm egg disintegration process. However, if concurrent diagnosis of *Strongyloides stercoralis* is envisaged, for example using the Baermann or Koga agar plate method [24], , freezing or storing samples on ice is not recommendable, since *S. stercoralis* larvae present in stool are temperature-sensitive [38].

Our study is an important contribution to the broad field of schistosomiasis and soil-transmitted helminthiasis diagnosis. In particular, our findings that keeping stool samples on ice or covered with a moist tissue delays hookworm egg disintegration and the observation of a decreased variance through stool homogenization on the FECs of *S. mansoni* without impacting on soil-transmitted helminth eggs are worth highlighting. Introducing these simple measures will help to achieve more accurate estimations of FECs and infection intensities in helminth diagnosis. Together with the random spatial distribution of *S. mansoni*, hookworm, and *T. trichiura* eggs within entire stool samples, these observations should be taken into consideration for stool transport, storage, and examination in laboratories of travel clinics in the EU and the US as well as for hospitals, and epidemiological studies, clinical trials, and helminth control programs in endemic settings.

## Supporting Information

Alternative Language Abstract S1 **Translation of the Abstract into German by Stefanie J. Krauth.**
(DOC)

Alternative Language Abstract S2 **Translation of the Abstract into French by Jean T. Coulibaly.**
(DOC)
