# Supplementary material for: An In-Depth Analysis of a Piece of Shit: Distribution of Schistosoma mansoni and Hookworm Eggs in Human Stool
Source: PLoS Negl Trop Dis. 2012 Dec 20;6(12):e1969. doi: 10.1371/journal.pntd.0001969 (PMC3527364; doi:10.1371/journal.pntd.0001969)
Supplement: Alternative Language Abstract S1 — Translation of the Abstract into German by Stefanie J. Krauth. (DOC) [file pntd.0001969.s001.doc]

**Tiefenanalyse eines Stücks Scheisse: Verteilung von *Schistosoma mansoni*- und Hakenwurmeiern in menschlichem Stuhl**

**Zusammenfassung**

***Hintergrund:*** Eine akkurate Diagnose von Helmintheninfektionen ist wichtig für die optimale Behandlung von Patienten. Es gibt jedoch eine beträchtliche Variation der Eizahlen innerhalb einzelner und zwischen verschiedenen Stuhlproben von Menschen. Das Homogenisieren von Stuhlproben wurde vorgeschlagen, um die Präzision der Diagnose zu verbessern, genaue Untersuchungen hierzu liegen jedoch nicht vor. Ein schnelles Verschwinden von Hakenwurmeiern stellt ein weiteres Problem in epidemiologischen Studien dar. Wir untersuchten die räumliche Verteiling von *Schistomsoma mansoni*- und Hakenwurmeiern in frischen Stuhlproben, den Effekt der Homogenisierung auf die Diagnose und bestimmten die Helmintheneizahlen in Stuhlproben welche über mehrere Stunden unter verschiedenen Umweltbedingungen aufbewahrt wurden.

***Methodologie:*** Vollständige Stuhlproben von 222 Individuen einer Ländlichen Gegend im Süden der Côte d’Ivoire wurden gesammelt und detailliert untersucht. Die Proben wurden in vier Stücke geteilt und die Verteilung der Helmintheneier vom vorderen Teil des Stuhls zum hinteren, sowie vom inneren zum äusseren untersucht. Einige der Proben wurden homogenisiert und die Eizahlen vor und nach der Homogenisierung wurden verglichen. Ausserdem untersuchten wir den Effekt verschiedener Aufbewahrungsmethoden und der Aufbewahrungsdauer auf die Eizahlen. Dabei wurde die normale Aufbewahrung im Schatten mit der Aufbewahrung auf Eis und dem Abdecken der Stuhlproben mit einem wassergetränkten Papiertuch verglichen.

***Wichtigste Ergebnisse:*** Wir fanden keine eindeutigen räumlichen Verteilungsmuster für *S. mansoni*- und Hakenwurmeier in Stuhlproben. Homogenisierung verminderte die *S. mansoni* Eizahlen (p = 0.026), während keine Auswirkung auf Hakenwürmer und andere mittels Boden-übertragenen Helminthen festgestellt werden konnte. Hakenwurm Eizahlen nahmen mit fortlaufender Zeit ab wohingegen das Aufbewahren der Stuhlproben auf Eis oder bedeckt mit einem feuchten Papiertuch, diese Abnahme verlangsamte (p < 0.005).

***Schlussfolgerung/Signifikanz:*** Unsere Ergebnisse haben wichtige Implikationen für die Diagnose von Helmintheninfektionen sowie für epidemiologische Studien, die Evaluation von Medikamentenstudien und die Überwachung von Kontrollprogrammen. Insbesondere empfehlen wir das Homogenisieren von Stuhlproben für eine akkurate Diagnose von *S. mansoni* Eiern während das Kühlen und Feuchthalten der Stuhlproben den Zerfall von Hakenwurmeiern verlangsamen konnte.

***Übersetzung:*** Stefanie J. Krauth
