# Supplementary material for: An In-Depth Analysis of a Piece of Shit: Distribution of Schistosoma mansoni and Hookworm Eggs in Human Stool
Source: PLoS Negl Trop Dis. 2012 Dec 20;6(12):e1969. doi: 10.1371/journal.pntd.0001969 (PMC3527364; doi:10.1371/journal.pntd.0001969)
Supplement: Alternative Language Abstract S2 — Translation of the Abstract into French by Jean T. Coulibaly. (DOC) [file pntd.0001969.s002.doc]

**Une Aalyse mnutieuse d’une Pièce de Merde: Répartition des Oeufs de *Schistosoma mansoni* et d’Ankylostosme dans les Selles Humaines.**

**Résumé**

***Etat des connaissances:*** Un diagnostic fiable des infections à helminths est important pour améliorer la prise en charge des patients. Cependant, il y a une considérable variation inter et intra spécimen du nombre d’œufs d’helminthe dans les matières fécale humaines. L’homogénéisation des échantillons de selles a été suggérée afin d’améliorer la fiabilité du diagnostic, mais il n y a pas assez investigations focalisées sur une étude détaillée des échantillons de selles. La désintégration rapide des œufs d’ankylostome constitue un autre problème dans les études épidémiologiques. Nous avons étudié la distribution spatiale des œufs de *Schistosoma mansoni* et d’ankylostome dans les échantillons de selles, l’effet de l’homogénéisation, et déterminé l’effet du temps sur nombre d’œufs dans les selles conservées sous différentes conditions.

***Méthodologie:*** Des échantillons entiers de selles ont été collectés chez 222 individus dans une zone rurale au sud de la Côte d’Ivoire. Les échantillons ont été sectionnés en quatre pièces et la localisation des œufs d’helminthes d’avant en arrière et de la surface au centre des différentes pièces ont été analysés. Certains échantillons ont été homogénéisés et le nombre d’oeufs dans les matières fécales comparé avant et après homogénéisation. L’effet des méthodes de conservation dans le temps sur le nombre d’oeufs compté dans les selles a été investigué, comparant les échantillons de selles, conservées dans la glace, couvert avec un tissu trempé dans de l’eau, ou gardés à l’ombre.

***Principaux résultats:*** Nous n’avons pas trouvé une distribution spatiale spécifique des oeufs de *S. mansoni* et d’ankylostome dans les échantillons de selles. L’homogénéisation diminue le nombre d’oeufs comptés de *S. mansoni* dans les selles (p = 0.026), bien qu’aucun effet n’a été observé pour le nombre d’œufs d’ankylostomes et de géohelminthes. Le nombre d’œufs comptés d’ankylostomes diminue avec le temps. La conservation des échantillons de selles sur la glace ou couvert avec un tissue trempé dans de l’eau ralentie la désintégration des œufs d’ankylostome (p <0.005).

***Conclusions/Portée:*** Nos résultats présentent d’importantes implications pour le diagnostic des helminthes au niveau de la prise en charge du patient et pour les études épidémiologiques, pour les études de l’efficacité des médicaments anthelminthiques et le suivi des programmes de contrôle. Particulièrement, l’homogénéisation des échantillons de selles est recommandée pour une détection fiable des œufs de *S. mansoni*, bien que maintenant les échantillons de selles collectés fraîches et surtout retardant la désintégration des œufs d’ankylostome.

***Traducteur:*** Jean T. Coulibaly
